# The Role of Noninvasive Ventilation in Patients with “Do Not Intubate” Order in the Emergency Setting

**DOI:** 10.1371/journal.pone.0149649

**Published:** 2016-02-22

**Authors:** Marta Vilaça, Irene Aragão, Teresa Cardoso, Cláudia Dias, Glória Cabral-Campello

**Affiliations:** 1 Medicine Integrated Master (MIM), Instituto de Ciências Biomédicas Abel Salazar (ICBAS), Oporto University (UP), Porto, Portugal; 2 Intensive Care Unit (UCIP), Oporto Hospital Center, Porto, Portugal; 3 Center for Health Technology and Services Research (CINTESIS) and Information Sciences and Decision on Health Department (CIDES), Faculty of Medicine, Oporto University (UP), Porto, Portugal; University of Bari, ITALY

## Abstract

**Background:**

Noninvasive ventilation (NIV) is being used increasingly in patients who have a “do not intubate” (DNI) order. However, the impact of NIV on the clinical and health-related quality of life (HRQOL) in the emergency setting is not known, nor is its effectiveness for relieving symptoms in end-of-life care.

**Objective:**

The aim of this prospective study was to determine the outcome and HRQOL impact of regular use of NIV outcomes on patients with a DNI order who were admitted to the emergency room department (ED). Methods: Eligible for participation were DNI-status patients who receive NIV for acute or acute-on-chronic respiratory failure when admitted to the ED of a tertiary care, university-affiliated, 600-bed hospital between January 2014 and December 2014. Patients were divided into 2 groups: (1) those whose DNI order related to a decision to withhold therapy and (2) those for whom any treatment, including NIV, was provided for symptom relief only. HRQOL was evaluated only in group 1, using the 12-item Short Form Health Survey (SF-12). Long-term outcome was evaluated 90 days after hospital discharge by means of a telephone interview.

**Results:**

During the study period 1727 patients were admitted to the ED, 243 were submitted to NIV and 70 (29%) were included in the study. Twenty-nine (41%) of the 70 enrollees received NIV for symptom relief only (group2). Active cancer [7% vs 35%, p = 0,004] and neuromuscular diseases [0% vs. 17%] were more prevalent in this group. NIV was stopped in 59% of the patients in group 2 due to lake of clinical benefit. The in-hospital mortality rate was 37% for group 1 and 86% for group 2 0,001). Among patients who were discharged from hospital, 23% of the group 1 and all patients in group 2 died within 90 days. Relative to baseline, no significant decline in HRQOL occurred in group 1 by 90 days postdischarge.

**Conclusion:**

The survival rate was 49% among DNI-status patients for whom NIV was used as a treatment in ED, and these patients did not experience a decline in HRQOL throughout the study. NIV did not provide significant relief of symptoms in more than half the patients who receive it for that purpose.

## Introduction

Noninvasive ventilation (NIV) is being used increasingly to support patients with a “do not intubate” (DNI) order who underwent respiratory failure and dyspnea [1–3]. A variety of factors may drive patients to the acute-care setting when nearing the end of life. Dyspnea is one of the most distressing symptoms experienced by dying patients, and it is a common reason for such patients to seek emergency care [4]. Many underlying disease states and acute illnesses cause shortness of breath at the end of life, and management tends to be symptomatic rather than diagnostic. In this context, invasive mechanical ventilation (IMV) is considered a nonbeneficial intervention that deprives patients of their dignity and ability to recognize their family members [5–8]. NIV does not have these disadvantages and may be more effective than oxygen alone for reducing dyspnea and decreasing the doses of morphine needed in patients with end-stage cancer [3].

In addition, the use of NIV for patients with DNI orders and potentially reversible causes of respiratory failure has become more common. Among such patients, it appears that those with reversible causes of dyspnea benefit from NIV used as a life-support intervention, with no decrease in health-related quality of life (HRQOL) and no increase in anxiety, depression or symptoms related to posttraumatic stress disorder after hospital discharge [6]. However, to our knowledge, all studies that included patients with DNI order were conducted in intensive care units (ICU) setting, and many of them did not differentiate the patients who received NIV with intention to treat from the patients in palliative care who received NIV for symptom relief [6,9]. Moreover, it appears that no study has evaluated the impact of NIV on HRQOL in patients with DNI order treated in non-ICU settings.

The aim of the present study was (1) to evaluate the impact of NIV on outcome and HRQOL in patients with DNI order who were admitted to the emergency department (ED) and (2) to assess whether NIV is effective for relieving symptoms in patients receiving palliative care only.

## Methods

Eligible for participation in this prospective study were all patients admitted to the ED of a tertiary care, 600-bed, university-affiliated hospital between January 2014 and December 2014, who underwent NIV for acute respiratory failure (ARF) or acute on chronic respiratory failure (ACRF).

The study was approved by the Hospital Ethics Committee and the Administration Board of Oporto Hospital Center (reference number 2014.075(051-DEFI/065-CES). Informed consent was waived for who died shortly after admission and the Hospital Ethics Committee approved the lack of informed consent for those patients. Written informed consent was obtained from all patients who were alive on the fifth to seventh days of admission, in accordance with the Ethics Committee (The written consent form was approved by this Committee). Collected data were anonymized.

In our hospital the Intensive Care Department is responsible for the ICU, high-dependency units (HDU) and the ED (located in the Accidents and Emergency Department). Only physicians with training in intensive care are permitted to work in these locations. The use of NIV for acute care is initiated only in these environments, and only patients who require chronic NIV are admitted to these wards.

Specific inclusion criteria were adult age, DNI order, and use of NIV for ARF or ACRF. Patients younger than 18 years of age were excluded. Patients with a DNI order were defined as those for whom healthcare professionals believed that tracheal intubation was not a therapeutic option.

The study population was divided in 2 groups. Group 1, the “*withholding* therapy decision” group, comprised patients for whom a decision was made by the attending physician not to start or increase a life-sustaining intervention [10] but who received NIV with survival hopes and the. Group 2, the “symptom relief only” group, comprise patients for whom only palliative care (comfort measures) was thought to be appropriate by the attending physician; these patients received NIV only to alleviate their dyspnea [9,11]. All treatment decisions were made jointly by an intensive care physician and the patient’s attending physician.

The study participants were subcategorized by the type of respiratory failure present at admission. Those with pH <7.35 and PaCO_2_> 45 mm Hg, associated with signs of respiratory distress (respiratory rate> 25 breaths / min, with the use of respiratory accessory muscles) were considered to have acute-on-chronic respiratory failure [12–16]. Those with PaO_2_ <60 mmHg or PaO_2_ / FiO_2_ <300, and PaCO_2_≤45mmHg, were classified as having acute hypoxemic respiratory failure [17–19].

HRQOL was evaluated at admission and 90 days after hospital discharge only for patients who received NIV as a treatment (group 1). The 12-intem Short Form Health Survey (SF-12) was used for this purpose. Within 5 to 7 days after admission patients were asked to completed this survey based on the 3 months preceding the admission [6,20]. Ninety days after hospital discharge, the surviving patients were interviewed by telephone and asked to complete the SF-12 questionnaire again to assess their current HRQOL. Patients who died during the 90-day postdischarge period were not included in the evaluation of long term HRQOL.

For all groups, length of stay in the ICU/HDU and hospital was documented, along with the in-hospital and 3 months mortality rates. Clinical variables and arterial blood gas levels were determined at baseline and 1 hour after initiation of NIV. Functional status was assessed using the Karnofsky performance scale, with ratings <70 representing patients who needed help with daily activities and ratings ≥70 denoting patients who were independent.

### Statistical analysis

Categorical variables were expressed as absolute and relative frequencies. Continuous variables were described as mean and standard deviation (SD) if normal distribution was present; otherwise, they were expressed as median and interquartile range (IQR).

To test hypotheses in groups of equal size, we used the t test for independent groups and the analysis of variance with 1 factor, when it was appropriate to assume normal distribution of variables. Testing of non-normal distribution of continuous variables was performed with nonparametric Mann-Whitney U or Kruskal-Wallis test, depending on the nature of the hypothesis being tested.

We assumed that a decrease of at least 20% in the SF-12 score represented clinical significance, in accordance with Azoulay et al [6].

For all hypotheses tested, the significance level was set at p = 0.05.

Data analysis was performed using the SPSS^®^ (version 20.0, Statistical Package for Social Sciences; IBM, Armonk, NY).

## Results

**Fig 1:**
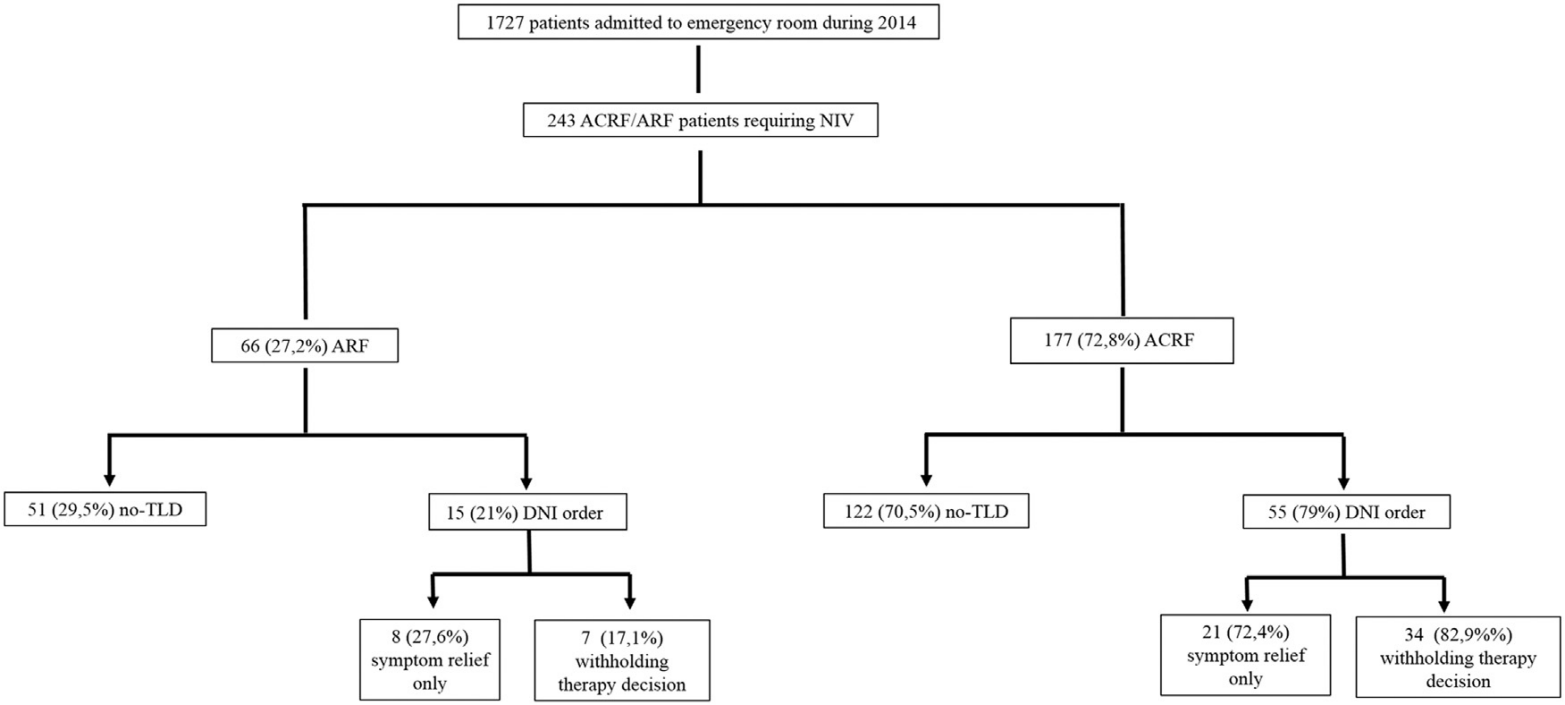
Flow chart of the study.

Between January 2014 and December 2014, 1727 patients were admitted to the ED, 243 of whom received NIV for acute or acute-on-chronic respiratory failure. Seventy (28.8%) of those 243 patients had a DNI order and were entered into the study. (Fig 1)

Of the 70 enrolls, 29 (41.4%) received NIV for symptom relief only (group 2), and 41 (58.6%) received it as treatment (group 1). The clinical characteristics of both groups are present in Table 1. There were no significant differences in demographic or clinical characteristics between the study groups, with exception of active cancer and neuromuscular disease, which were more prevalent in group 2 (Table 1).

**Table 1:** Characteristics of Patients with DNI order who received NIV.

Characteristics	Group 1: NIV treatment (n = 41)	Group 2: Symptoms relief only (n = 29)	p^a^
Age in years, median (IQR)	82.0 (75.0–87.0)	79.0 (73.0–85.0)	0.299
Male gender, n (%)	23 (56.1%)	18 (62.1%)	0.617
Type of respiratory failure, n (%)			0.291
ACRF	34 (82.9%)	21 72.4%)	
ARF	7 (17.1%)	8 (27.6%)	
Cause of respiratory failure, n (%)			
COPD exacerbation	22 (53.7%)	9 (31.0%)	0.060
ACPE	15 (36.6%)	6 (20.7%)	0.153
Pneumonia	11 (26.8%)	11 (37.9%)	0.324
Sepsis	0 (0.0%)	2 (6.9%)	0.168^b^
Others	14 (34.1%)	3 (10.3%)	0.261^b^
Co-morbidities, n (%)			
Congestive heart failure (NYHA)	19 (46.3%)	9 (31.0%)	0.198
COPD	21 (51.2%)	9 (31.0%)	0.093
Active cancer (all types)	3 (7.3%)	10 (34.5%)	0.004
Neuromuscular diseases	0 (0.0%)	5 (17.2%)	0.010^b^
Karnofsky performance rating, n (%)			0.952
≥70	13 (31.7%)	9 (31.0%)	-
<70	28 (68.3%)	20 (69.0%)	-

ACPE, acute cardiogenic pulmonary edema; ACRF, acute-on-chronic respiratory failure; ARF, acute respiratory failure; COPD, chronic obstructive pulmonary disease; DNI, “do not intubate”; NIV, noninvasive ventilation; NYHA, New York Heart Association.

^a^ According to Chi square test unless otherwise indicated.

^b^ According to Fisher's exact test.

More than two-thirds of each group had lower functional status (Karnofsky rating <70). Due to lack of clinical benefits, NIV was stopped in 19.5% of patients in group 1 an in 58.6% in group 2 (p<0.001). Almost all patients included in group 2 died in the first day of admission, and none was admitted to the ICU or HDU. Twenty-three (56%) of the patients in group 1 were admitted to the ICU or HDU. The in-hospital mortality rate was 36.6% in group 1and 86.2% in group 2. Moreover, 63.4% of the patients in group 1 were alive at discharge, 77% of whom remained alive 90 days after discharge. No patient from group 2 was alive 90 days after discharge (Table 2).

**Table 2:** Outcomes of the Patients with DNI order who received NIV.

Outcome	Group 1:NIV treatment (n = 41)	Group 2:Symptom relief only (n = 29)	P^a^
Stop NIV, n (%)	8 (19.5%)	17 (58.6%)	<0.001
Days in ICU/HDU, median (IQR)	3 (0–7)	0 (0–0)	<0.001
Days in hospital, median (IQR)	10 (5–17)	1 (0–5)	<0.001
Died in hospital, n (%)	15 (36.6%)	25 (86.2%)	<0.001
Died within 90 days of discharge, n (%)	6 (23.0%)^c^	4 (100%)^c^	0.009^b^
SF-12 at admission, median (IQR)	30 (27–31) (n = 17)	-	
SF- 12 on day 90 after hospital discharge, median (IQR)	27 (26–32) (n = 17)	-	

DNI, “do not intubate”; ICU, intensive care unit; HDU, high dependency unit; IQR, interquartile range; NIV, noninvasive ventilation.

^a^ According to Chi square test unless otherwise indicated.

^b^ According to Fisher's exact test.

^c^ These percentages are based on the number of patients discharged: n = 26 for group 1; n = 4 for group 2.

Among the 41 patients included group 1, 26 (63%) were alive at hospital discharge, and 20 (49%) were alive 90 days later. Seventeen (85%) of the 20 respond to the SF-12, allowing evaluation of HRQOL.

The interviews were conducted between 90 and 162 days after discharge (median, 104 days). Most (15%) of the patients who did not participate in the follow-up interview either declined the invitation or did not answer the telephone.

**Fig 2:**
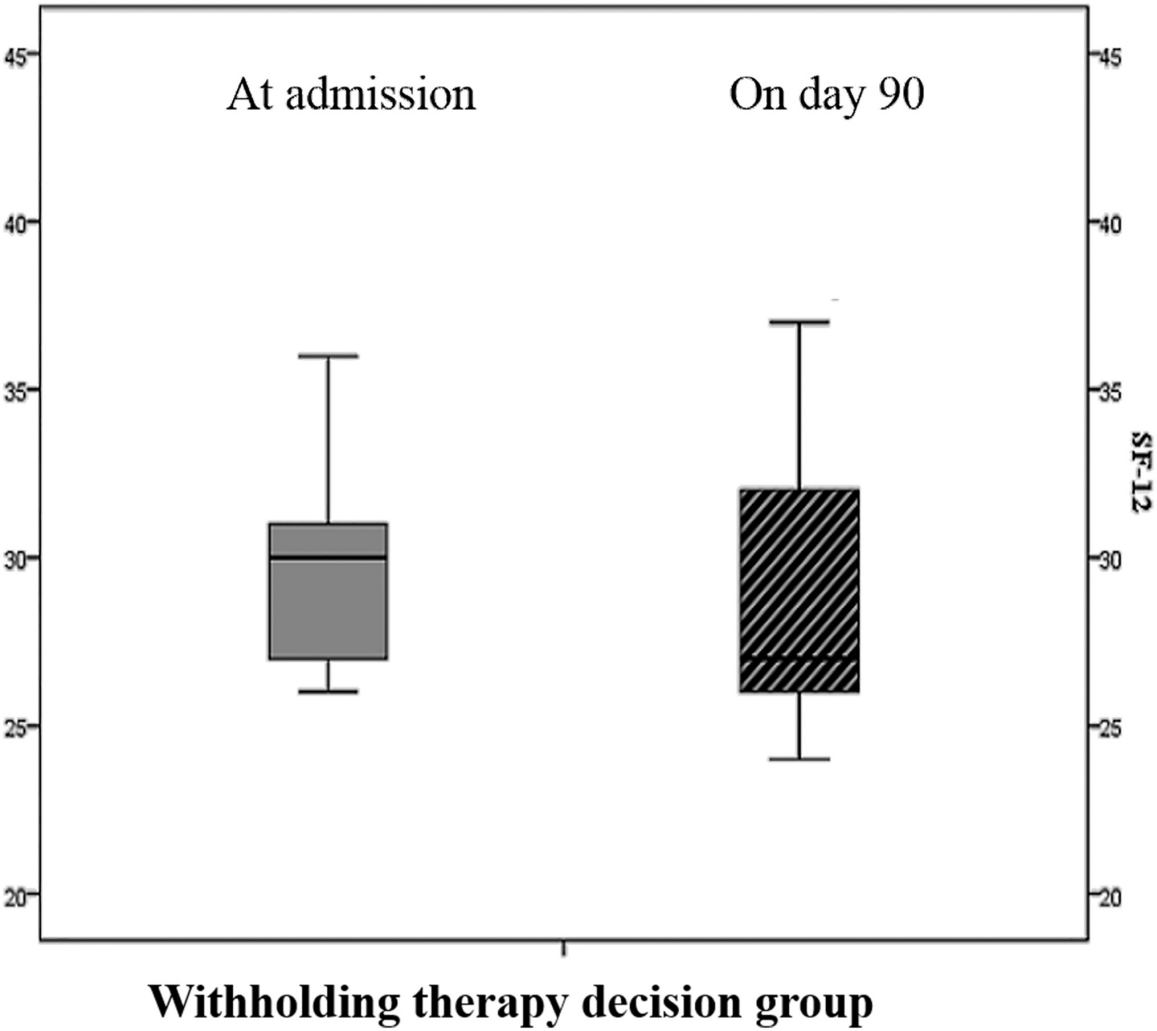
HRQOL at admission and at day- 90 postdischarge in patients with a DNI order.

In group 1, no significant difference was observed between HRQOL at admission and at day-90 postdischarge. (Table 2 *and* Fig 2)

## Discussion

Several important findings from our study. We observed that DNI status was defined for nearly one-third of patients admitted to the ED who received NIV. Patients with a DNI order who were alive 90 days after hospital discharge experienced no significant decline in HRQOL from baseline (admission). Long-term (90-day) follow-up demonstrated a survival rate of 49% in group 1 (NIV used as treatment). In patients under palliative care, who represent 40% of all study patients with DNI order, NIV was stopped in more than 50% due to lack of symptom improvement.

The use of NIV in non-ICU settings has been increasing despite the lack of evidence of its safety and efficacy outside the ICU. [21–24]. However, Cabrini et al [25] demonstrated that, under the supervision of a medical emergency team, NIV could be applied in a variety of non-ICU settings, with a high success rate and few complications [25]. Our study was conducted in the ED of a tertiary care, university-affiliated hospital, which is supervised by the ICU team (doctor and nurse) and provides continuous clinical monitoring.

A unique aspect or our study is the grouping of patients by whether NIV was performed with an intent to treat or solely for symptom relief. To our knowledge, this distinction has not been made in other studies. Azoulay et al [6], in a multicenter prospective observational study in the ICU setting, demonstrated that DNI order was present for one-fifth of ICU patients who received NIV, which is lower than the prevalence in our study [6]. This likely can be explained by the difference in study setting (ours was in ED). Only 56% of the patients in group 1 of our study (withholding therapy decision) were admitted to the ICU or HDU; therefore, the difference in DNI-order prevalence may be attributable to selection bias. Four our DNI-status patients who received NIV as treatment (group 1: withholding therapy), the in-hospital mortality rate was similar to that found by Azoulay et al [6].

Another important finding of our study is the fact that half the patients in group 1 were alive 90 days after hospital discharge, with no significant change in HRQOL since the time of admission. Thus, we agree with Azoulay et al [6] that NIV appears to be valuable treatment for ARF or ACRF in patients who do not benefit from IMV but who may benefit from other life-supporting interventions [6].

In our study, 41% the patients with a DNI order admitted to the ED received NIV for symptom relief only (group 2), and only 4 (14%) of these were alive at discharge. Most patients in group 2 died on the day of hospital admission, and NIV had to be stopped in more than half of group 2, which suggests that NIV may not be an effective palliative measure in such patients. This finding differs from results of a study by Nava et al [22], who found that NIV was more effective than oxygen in reducing the dyspnea, and that the dose of morphine could be decreased in patients with end-stage cancer [23]. Therefore, future research is warranted to assess the effects of NIV as comfort therapy only, including the perceptions and satisfaction of family members and the perspectives of healthcare professionals. This study has several limitations. Because it is a single-center study, conducted in a tertiary care hospital with extensive NIV experience, [18], the results may not be generalizable to other settings or circumstances, and may represent underestimations or overestimations of actual data obtained in different settings or larger populations. Our study population was small, and became even smaller when the patients were subcategorized, many of whom did not survive through 90 days postdischarge. Therefore, the results must be interpreted with caution. Only half the patients in group 1 were alive90 days after discharge, which result in a small sample for evaluating HRQOL. However, the interview response rate was high (85%) among these survivors.

## Conclusion

In the present study, a DNI order was present for nearly one-third of ED-admitted patients who received NIV for ARF or ACRF. Nearly half the DNI-status who received NIV as a treatment were alive 90 days after hospital discharge, none of whom experienced a noteworthy change in HRQOL throughout the study. Among patients who received NIV only for symptom relief, more than half did not experience significant improvement of symptoms. All patients in this group died within 90 days of discharge. Additional research on this topic is warranted, particularly larger studies of NIV in patients receiving palliative care.

## Supporting Information

S1 Table Minimum Data Set. (PDF)
