# Supplementary material for: The Role of Noninvasive Ventilation in Patients with “Do Not Intubate” Order in the Emergency Setting
Source: PLoS One. 2016 Feb 22;11(2):e0149649. doi: 10.1371/journal.pone.0149649 (PMC4763309; doi:10.1371/journal.pone.0149649)
Supplement: S1 Table — (PDF) [file pone.0149649.s001.pdf]

Gender	Age	GCSAtAdmition	DNI order group	COPDExacerbation	ACPE
F	93	4	Withholding therapy decision	yes	
F	88	15	Withholding therapy decision		yes
M	87	3	Withholding therapy decision	yes	
M	92	4	Withholding therapy decision	yes	
F	74	9	Withholding therapy decision	yes	
M	49	11	Symptoms relief only		
F	88	14	Withholding therapy decision		
M	62	14	Withholding therapy decision	yes	
M	83	10	Withholding therapy decision		
F	83	3	Symptoms relief only		
M	47	14	Withholding therapy decision		
M	47	8	Symptoms relief only		yes
M	68	13	Symptoms relief only	yes	
M	80	15	Symptoms relief only		yes
F	77	8	Withholding therapy decision		
M	78	3	Symptoms relief only		
M	85	15	Symptoms relief only		
M	64	15	Symptoms relief only		
M	86	13	Symptoms relief only		yes
F	78	3	Withholding therapy decision		
F	88	9	Symptoms relief only		
M	86	3	Symptoms relief only		
M	38	3	Symptoms relief only	yes	
F	80	15	Symptoms relief only		
M	89	10	Symptoms relief only		yes
F	88	15	Withholding therapy decision		
M	77	9	Symptoms relief only		
F	79	8	Symptoms relief only		
M	57	15	Symptoms relief only		
F	94	14	Symptoms relief only		
F	88	15	Withholding therapy decision		yes
M	86	15	Withholding therapy decision	yes	yes
F	79	13	Withholding therapy decision		
M	86	3	Symptoms relief only	yes	
F	95	7	Symptoms relief only		yes
M	94	9	Withholding therapy decision		yes
F	86	15	Symptoms relief only		
F	85	8	Symptoms relief only		yes
M	63	15	Withholding therapy decision		
M	25	15	Symptoms relief only		
M	74	15	Withholding therapy decision		yes
M	75	14	Symptoms relief only	yes	
F	99	10	Symptoms relief only	yes	
M	73	8	Symptoms relief only		
F	91	15	Withholding therapy decision		yes
M	79	10	Withholding therapy decision		yes
M	87	15	Withholding therapy decision		yes
M	84	15	Withholding therapy decision		yes
F	88	15	Withholding therapy decision	yes	

F	85	7	Symptoms relief only	yes	
M	75	15	Symptoms relief only	yes	
F	86	15	Withholding therapy decision		yes
M	79	15	Withholding therapy decision		yes
F	75	15	Withholding therapy decision		yes
M	83	15	Withholding therapy decision		
M	82	14	Withholding therapy decision	yes	
M	68	15	Withholding therapy decision	yes	
M	68	15	Withholding therapy decision		
M	81	15	Withholding therapy decision	yes	
F	77	6	Withholding therapy decision		
F	38	3	Symptoms relief only		
F	90	15	Withholding therapy decision		yes
F	90	7	Withholding therapy decision		
M	77	3	Symptoms relief only		
M	77	15	Withholding therapy decision		
M	75	15	Withholding therapy decision	yes	
M	62	15	Withholding therapy decision	yes	
F	92	15	Withholding therapy decision		
F	89	15	Withholding therapy decision		yes
M	82	15	Withholding therapy decision		yes
M	75	15	Withholding therapy decision		

Pneumonia	Sepsis	Others	CongestiveHeartFailure	ActiveCancer	COPD	NeuromuscularDiseses
		yes			yes	
		yes			yes	
		yes	yes			
		yes				yes
		yes	yes			
			yes		yes	
yes					yes	
yes						
		yes			yes	
					yes	
			yes		yes	
yes		yes	yes		yes	
yes				yes		
yes				yes	yes	
yes						yes
				yes		
yes			yes			
yes	yes					
yes	yes				yes	
		yes				yes
			yes			
		yes	yes			
						yes
yes						
				yes		
			yes		yes	
yes						
			yes		yes	
			yes	yes		
			yes		yes	
yes			yes	yes	yes	
			yes	yes		
				yes	yes	
				yes		
			yes			
yes				yes	yes	
			yes		yes	
			yes			
yes			yes			
		yes			yes	

yes		yes	yes			
			yes	yes	yes	
			yes			
			yes	yes		
		yes	yes		yes	
			yes		yes	
					yes	
yes						
yes			yes	yes		
yes					yes	
						yes
yes		yes	yes			
yes						
yes	yes				yes	
yes					yes	
					yes	
		yes				
			yes			
			yes		yes	
yes					yes	

Karnofskyscale	VNIStop	InHospitalMoratlity	ICUStay	DaysSpendinHospital	Day90Mortality
<70		yes	0	0	
>=70		no	7	13	no
<70	yes	yes	0	0	
<70	yes	yes	0	0	
<70		no	0	16	no
<70		yes	0	0	
<70		yes	7	7	
<70	yes	yes	32	98	
<70	yes	yes	0	0	
<70		yes	0	0	
>=70		no	3	18	no
>=70	yes	yes	0	7	
>=70		no	0	10	no
<70	yes	yes	0	4	
>=70		no	0	30	no
<70	yes	yes	0	0	
>=70	yes	yes	0	0	
<70	yes	yes	0	15	
<70	yes	yes	0	0	
<70		no	4	37	no
<70	yes	yes	0	0	
>=70	yes	yes	0	0	
>=70	yes	yes	0	0	
>=70	yes	yes	0	0	
>=70		yes	0	0	
<70	yes	no	0	9	yes
<70	yes	yes	0	0	
<70		yes	0	0	
<70	yes	yes	0	0	
>=70		no	0	11	yes
>=70		no	0	3	yes
<70	yes	yes	0	0	
>=70	yes	yes	5	5	
<70		yes	0	15	
<70		yes	0	5	
<70		no	6	10	yes
<70	yes	yes	0	0	
<70	2	yes	0	2	
<70	yes	yes	3	3	
>=70	yes	yes	0	0	
>=70		no	5	12	no
<70		no	0	9	yes
<70		no	0	5	yes
<70		yes	0	1	
<70		no	6	10	yes
>=70		yes	0	10	
>=70		no	3	27	yes
<70		yes	15	35	
>=70		no	3	18	no

<70	yes	yes	0	2	
<70		no	0	3	yes
<70		no	0	4	no
>=70		no	0	6	no
<70		no	0	2	no
>=70		yes	10	15	
<70		no	4	9	no
<70		no	7	54	
<70		yes	17	17	
>=70		yes	0	8	
<70		no	7	10	no
<70		yes	0	0	
<70		no	0	34	yes
<70		no	9	17	no
>=70	yes	yes	0	4	
>=70		no	16	28	no
<70		no	12	17	no
<70		no	5	11	no
<70		no	0	19	no
<70		yes	8	17	
<70		no	0	1	no
<70		no	0	7	no

SF12AtAdmition	SF12OnDay90
30	33
26	25
43	37
32	26
36	36
30	30
26	29
29	26

34	32
26	25
30	34
27	27
27	27
34	30
28	28
31	24
30	27
26	24
